# Age-related slowing of response selection and production in a visual choice reaction time task

**DOI:** 10.3389/fnhum.2015.00193

**Published:** 2015-04-23

**Authors:** David L. Woods, John M. Wyma, E. William Yund, Timothy J. Herron, Bruce Reed

**Affiliations:** ^1^Human Cognitive Neurophysiology Laboratory, Veterans Affairs Northern California Health Care SystemMartinez, CA, USA; ^2^The Department of Neurology, University of California DavisSacramento, CA, USA; ^3^Center for Neurosciences, University of California DavisCA, USA; ^4^Center for Mind and Brain, University of California DavisCA, USA; ^5^Alzheimer’s Disease Center, Department of Neurology, University of California DavisCA, USA

**Keywords:** aging, timing, processing speed, motor, handedness, hemisphere, replication, executive function

## Abstract

Aging is associated with delayed processing in choice reaction time (CRT) tasks, but the processing stages most impacted by aging have not been clearly identified. Here, we analyzed CRT latencies in a computerized serial visual feature-conjunction task. Participants responded to a target letter (probability 40%) by pressing one mouse button, and responded to distractor letters differing either in color, shape, or both features from the target (probabilities 20% each) by pressing the other mouse button. Stimuli were presented randomly to the left and right visual fields and stimulus onset asynchronies (SOAs) were adaptively reduced following correct responses using a staircase procedure. In Experiment 1, we tested 1466 participants who ranged in age from 18 to 65 years. CRT latencies increased significantly with age (*r* = 0.47, 2.80 ms/year). Central processing time (CPT), isolated by subtracting simple reaction times (SRT) (obtained in a companion experiment performed on the same day) from CRT latencies, accounted for more than 80% of age-related CRT slowing, with most of the remaining increase in latency due to slowed motor responses. Participants were faster and more accurate when the stimulus location was spatially compatible with the mouse button used for responding, and this effect increased slightly with age. Participants took longer to respond to distractors with target color or shape than to distractors with no target features. However, the additional time needed to discriminate the more target-like distractors did not increase with age. In Experiment 2, we replicated the findings of Experiment 1 in a second population of 178 participants (ages 18–82 years). CRT latencies did not differ significantly in the two experiments, and similar effects of age, distractor similarity, and stimulus-response spatial compatibility were found. The results suggest that the age-related slowing in visual CRT latencies is largely due to delays in response selection and production.

## Introduction: Experiment 1

Visual choice reaction time (CRT) tasks have been widely used to measure age-related declines in processing speed (Salthouse, 2000; Anstey et al., 2005; Deary and Der, 2005; Bugg et al., 2006; Der and Deary, 2006; Deary et al., 2010, 2011; Godefroy et al., 2010; Ballesteros et al., 2013). Previous studies have found significant age-related slowing of visual CRT latencies in a variety of experiments, including paradigms with two stimuli and two response buttons (Bugg et al., 2006; Feeney et al., 2013), paradigms with four stimuli and two response buttons, and paradigms with four stimuli and four response buttons (Deary et al., 2011). Table 1 provides a summary of recent large-scale CRT studies. In all of the studies, CRT latencies are minimal in young adulthood and increase by 2.0–3.4 ms for each year of age thereafter.

**Table 1:** **Large-scale studies of age-related changes in visual choice reaction time**.

Study	*N*	Age range	CRT (ms)	SD (ms)	ISSD (ms)	CV (%)	Age slope ms/yr	SOA	No. trials
Bugg et al. (2006)	196	20–89	497	91				1–5 s	32 (0)
Dykiert et al. (2012b)	312	18–59 (40)	518	68	88	17%	2.2	1–3 s	40 (8)
Vincent et al. (2012)	107,413	17–65 (27)	592	90			2.0	1–2 s	40 (0)
Deary et al. (2001)	900	(56)	728	108	131	18%
Deary et al. (2011) box	150	18–80 (48)	556	92	108	19%	2.5	1–3 s	40 (8)
Deary et al. (2011) PC	150	18–80 (48)	475	94	100	21%	2.5	1–3 s	40 (8)
Deary and Der (2005)	1,900	16–60	638	84	119	19%	3.4	1–3 s	40 (8)
Feeney et al. (2013)	4,453	47–78 (64)	801	192	301	38%	N/A	N/A	N/A
Experiment 1	1,466	18–65 (46)	550	72	162	30%	2.8	Ad.(0.52–2.5)	140
Experiment 2	178	18–82 (41)	546	79	170	31%	1.9	Ad.(0.58–2.5)	140

*N = number of participants. CRT = choice reaction time. ISSD = intrasubject standard deviation. CV = coefficient of variation. Age-slope = estimated increase in CRT latencies in ms/year. SOA = stimulus onset asynchrony. No. trials = number of trials with the number of practice trials in parentheses. Ad. = adaptive based on performance (minimal SOAs seen in different subjects are shown in parentheses)*.

CRT tasks engage a number of processing stages that may be affected by aging. Aging has been associated with declines in alertness and attention (Müller-Oehring et al., 2013), as well as slowed stimulus perception (Anstey et al., 2001; Glass, 2007) and discrimination (Madden and Allen, 1995; Schroeder et al., 1995; Yamaguchi et al., 1995). Aging also slows response selection due, in part, to increases in intrahemispheric (van der Lubbe and Verleger, 2002; Rabbitt et al., 2007) and transcallosal (Jeeves and Moes, 1996) transmission times between sensory and motor cortex. In addition, aging delays response generation in motor cortex (Falkenstein et al., 2006; Roggeveen et al., 2007), and responses are further slowed by age-related reductions in nerve conduction velocity (Li et al., 1998; Tobimatsu et al., 1998; Levin et al., 2011) and slowed muscle contraction (Lewis and Brown, 1994).

However, age-related changes are not uniform for different processing stages. Simple reaction time (SRT) latencies, which engage stimulus detection and response production stages, increase by 20–40 ms from age 20–65 (Woods et al., 2015). In contrast, CRT latencies, which include the additional processing stages of stimulus discrimination and response selection, slow by 90–120 ms over the same age range (see Table 1). Thus, age-related slowing in stimulus perception and motor responses would appear to account for only a small percentage of the age-related slowing of CRT latencies; i.e., most of the age-related slowing in CRT latencies would appear to reflect delays in the time needed either to discriminate stimuli or to select and execute the appropriate response.

Age-related changes in visual discrimination have been examined extensively in visual search tasks (Plude and Doussard-Roosevelt, 1989; Schialfa et al., 1998; Hommel et al., 2004). Older participants, like their younger counterparts, have flat search slopes as a function of display size in feature search tasks where targets are distinguished from distractors by color or shape (Plude and Doussard-Roosevelt, 1989). However, in feature-conjunction conditions, where targets are distinguished from distractors by a combination of features (e.g., color *and* shape), older participants show steeper search slopes. These results suggest that aging slows the more attentionally-demanding feature-integration stage of stimulus processing (Plude and Doussard-Roosevelt, 1989; Müller-Oehring et al., 2013).

Here, we analyzed CRT latencies in a population sample of 1466 adults ranging in age from 18–65 years using a serial feature-conjunction task. In order to optimize the utility of the normative data for subsequent clinical test applications, individual stimuli were presented serially to the left or right visual field and stimulus onset asynchronies (SOAs) were adaptively reduced based on participant accuracy. Participants pressed one mouse button in response to the target letter (a blue P, probability 40%), and pressed the other mouse button in response to distractor letters that differed from the target in color (orange P, 20%), shape (blue F, 20%), or both color and shape (orange F, 20%).

We expected to find significant age-related slowing because each trial required the participant to integrate color and shape information before choosing an appropriate response. We anticipated that CRT latencies would be faster for distractors with no target features than for distractors that shared either target color or shape, and that this difference would increase with age, reflecting an increase in sensory processing time (Habekost et al., 2013). Finally, we anticipated that participants would respond more rapidly when the stimulus and response button were spatially compatible, and that this spatial-compatibility effect would also increase with age (van der Lubbe and Verleger, 2002).

In order to clarify the processing stages affected by aging, we included estimates of stimulus detection time (SDT, the time needed to detect a visual stimulus), measured in the same participants in an SRT task (Woods et al., 2015), and movement initiation time (MIT, the time to depress the response button), which was obtained in the same participants in a speeded finger tapping task (Hubel et al., 2013a).

## Methods: Experiment 1

### Participants

We studied a subset of 1637 community volunteers in Rotorua, New Zealand, who participated in a study of the health effects of environmental exposure to varying levels of naturally-occurring hydrogen sulfide (H_2_S) (Reed et al., 2014). Written informed consent was obtained from all participants following Institutional Review Board study procedures for the University of California, Berkeley and the Northern Ethics Committee in New Zealand.

Because we wanted to analyze age-related changes in different processing stages through a comparison of results across tests, we eliminated 108 participants who lacked complete data sets in either a finger-tapping test (Hubel et al., 2013a), a SRT test (Woods et al., 2015), or who lacked complete data in all of the CRT test conditions. We also eliminated 41 ambidextrous participants whose finger tapping data had not been analyzed, and 22 participants who had unexplained poor SRT performance (SRT hit rates below 80%).

Of the remaining 1466 participants, 40.0% were men, 10.7% were left-handed by self-report (based on writing hand), and all were between the ages of 18 and 65 (mean age = 46.3 yrs. for men, 45.4 yrs. for women, see Table 2). They had an average United States equivalent of 12.6 years of education, including 76.7% with a secondary school qualification and 48.4% with additional education including a bachelor’s degree (12.1%), master’s degree (2.9%), doctorate (1.6%), or other trade, technical, or professional qualification (31.7%). Ethnically, the sample was primarily of European background (80.0%) and New Zealand Maori (15.6%). The remaining 4.4% represented a variety of ethnicities, none representing more than 1% of the sample. 78.7% of the sample was employed.

**Table 2:** **Performance of subjects in the seven different age groups in Experiment 1**.

	Experiment 1								Experiment 2
Ages	18–24	25–31	32–38	39–45	46–51	51–58	59–65	Total	Total
N	86	114	200	274	274	272	246	1466	178
Age	20.83	28.55	35.45	42.26	48.63	54.97	61.63	45.79	41.24
Edu	11.3	12.5	13.5	12.3	12.3	12.6	12.4	12.5	14.7
% male	34%	36%	42%	37%	41%	44%	40%	40%	56%
CRT	472	504	521	541	563	575	590	550	546
CRT SD	58.8	65.5	61.6	65.5	65.8	61.8	69.0	72.2	79.0
CPT	262	280	296	311	326	340	355	319	314
ISSD	144	159	160	162	163	163	170	162	170
CV	30.1%	31.1%	30.7%	29.9%	29.1%	28.4%	28.9%	29.5%	31.1%
mSOA	959	811	803	797	808	828	867	825	817
AR-CRT *z*	−0.12	0.04	−0.01	0.01	0.08	−0.01	−0.07	0.00	0.15
l-mSOA-*z*	0.74	−0.09	−0.12	−0.18	−0.10	0.03	0.16	0.00	−0.07
Omni-*z*	0.46	−0.04	−0.10	−0.13	−0.01	0.03	0.06	0.00	0.08
AR-CPT *z*	−0.15	0.06	0.00	0.03	0.07	−0.02	−0.07	0.00	0.10

*Also shown are the averages of all subjects in Experiments 1 and 2. CV = coefficient of variation. CPT = Central processing time (CRT-simple reaction time). mSOA = minimum SOA. AR-CRT = age-regressed CRT. L-mSOA-z = log-transformed mSOA z-score. Omni (omnibus) z = normalized sum of L-mSOA and AR-CRT z-scores. AR-CPT-z = age-regressed CPT value. The regressed z-scores were derived using parameters from Experiment 1. See Table 1 for additional abbreviations*.

### Stimuli and Task

**Figure 1:**
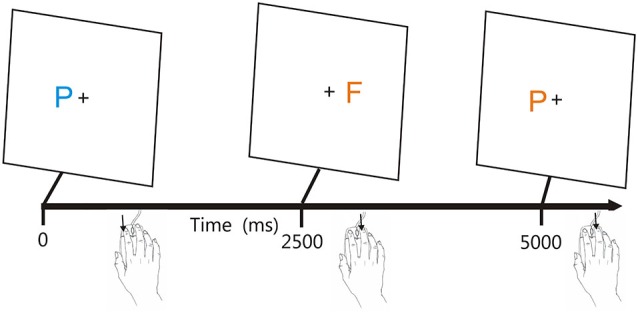
**The adaptive visual feature conjunction task**. Subjects performed a visual feature conjunction task with colored letters (blue P, blue F, orange P, or orange F) subtending 0.5° of visual angle randomly presented to the left or right hemifield, 1.6° from the fixation cross. Stimulus durations were 200 ms. Right-handed subjects pressed the left mouse button for targets (blue P’s, probability 40%) and pressed the right mouse button for non-targets, i.e., letters which resembled the target in color, shape, or neither feature (probability 20% each). Stimuli could occur ipsilateral (trials 1 and 2) or contralateral (trial 3) to the mouse button used for responding. Stimulus onset asynchronies (SOAs) were initially set at 2500 ms and were either reduced by 3% following each pair of successive hits or increased by 3% following each miss.

Figure 1 shows the stimuli. Participants responded to the target (blue P) by pressing the left mouse button, and responded to the other three stimuli with a right mouse button press, with responses reversed for participants who preferred to use the mouse with their left hand. The letters P and F appeared in blue or orange colors (selected to reduce the influence of possible dichromatic anomalies), with distractors differing from the target in both color and shape (orange F), only shape (blue F), or only color (orange P).

Stimuli were of high contrast (orange letters were 14.3 cd/m^2^ and blue letters were 3.5 cd/m^2^) and were presented on a bright background (40 cd/m^2^). Stimulus durations were fixed at 200 ms. Participants first responded to criterion levels (80% correct) in 20 practice trials, which were repeated if necessary. SOAs began at 2500 ms and were either reduced by 3% following two successive correct responses or increased by 3% following each error or response omission (miss). One-hundred-forty trials were included in the test. CRT testing required approximately 5 min, and occurred midway through a 30-min computerized test battery that included, in order, tests of finger tapping (Hubel et al., 2013a,b), SRT (Woods et al., 2015), CRT, digit span (Woods et al., 2011a,b), and paced auditory serial addition. Testing was performed in a quiet room using a standard Personal Computer (PC) controlled by Presentation® software (Versions 13 and 14, NeuroBehavioral Systems, Berkeley CA). Participants sat 0.7 m from a 17” Samsung Syncmaster Liquid-Crystal Display (LCD) computer monitor set at a 60 Hz refresh rate.

### Hardware and Software Calibration

Reaction time measurements are influenced by the computer hardware used for stimulus display and response monitoring (Plant and Turner, 2009; Neath et al., 2011). Therefore, measures of timing precision are necessary to compare results across different computer systems (Plant and Quinlan, 2013). Presentation® synchronizes stimulus delivery to the video refresh rate (e.g., stimuli were presented for 12 video frames at 60 Hz). We measured a delay of 11.0 ms in the illumination of the Samsung Syncmaster using a StimTracker (Cedrus, San Pedro, CA) photodiode. Responses were recorded with a high-precision gaming mouse (Razer, Copperhead, Carlsbad, CA) using an internal driver with a 1.0 kHz USB sampling rate, whose response latency was measured at 6.8 ms. Thus, hardware delays totaled 17.8 ms.

In addition to hardware delays, software interruptions can introduce unpredictable delays that increase CRT latencies and trial-to-trial latency variability. The frequency and duration of software interruptions depends on both the design of the stimulus-delivery software and on the number and type of extraneous software processes running concurrently. Timing interruptions must be continuously monitored throughout an experiment to assure timing precision. Presentation software reports event-time uncertainties for each event during an experiment by continuously sampling the 100 kHz programmable clock. CRT measurements were extremely precise: 252, 651 events showed a median event-time uncertainty of 0.16 ms (range 0.1–34.3 ms), with 99.9% of events showing timing uncertainties less than 1.04 ms.

### Data Analysis

We quantified mean CRT latencies for each type of stimulus, along with intrasubject (trial-to-trial) CRT standard deviations and hit rates. A response window of 250–1250 ms was used, and failure to generate a response during this interval was categorized as an omission. The minimum SOA (mSOA) was also measured for each participant. In cases where SOAs were reduced below 1250 ms, multiple responses could occur within a response window. In this case, responses were assigned to stimuli in the order in which they occurred.

### Statistical Analysis

Participants were classified into seven different 7 year wide age ranges (e.g., from 18–24 years to 59–65 years). The results were first analyzed using a multifactor mixed Analysis of Variance (ANOVA) with Age-group, Sex, Stimulus-type, SOA, and Hemifield (ipsilateral or contralateral to the responding hand) as independent variables. Separate ANOVAs were performed for mean CRT, Hit Rate, Intraparticipant CRT Standard Deviation, and Intraparticipant Coefficient of Variation. Greenhouse-Geisser corrections of degrees of freedom were uniformly used in computing *p* values in order to correct for covariation within factors or interactions. Effect sizes are reported as partial *ω*^2^ values. Correlation analysis was also used to analyze the effects of age and education, and to develop age-regression functions. SPSS was used to calculate 95% confidence intervals for correlation coefficients. Certain pairwise effects were also analyzed with Student’s *t*-tests, using a model that assumes unequal variance in the different participant groups when appropriate.

## Results: Experiment 1

**Figure 2:**
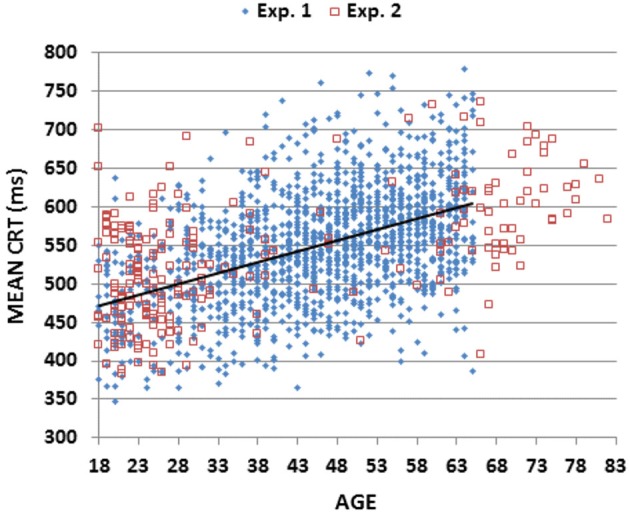
**Mean choice reaction times (CRTs)**. Mean CRTs averaged over stimulus types for subjects of different ages from Experiments 1 (blue diamonds) and 2 (open red squares). The linear fit for Experiment 1 data is shown.

Figure 2 (blue diamonds) shows a scatter plot of mean CRT latencies as a function of participant age, and Table 2 provides a summary of demographic information and performance data including CRT latencies and additional metrics that are described below. We first analyzed the results by Age-group with Visual Field and Type of stimulus (target, distractor with no target features, distractor with target color, and distractor with target shape) as factors. The effects of visual field were also analyzed.

The Age-Group effect was highly significant (*F*_(6,1462)_ = 62.94, *p* < 0.0001, partial *ω*^2^ = 0.20): CRT latencies increased with Age-group (see Table 2: from 476 ms in the youngest group to 595 ms in the oldest). All pairwise comparisons of adjacent Age-groups reached significance (*p* < 0.05 uncorrected), and power analysis showed a 99% probability of detecting an effect of aging at the *p* < 0.05 level in 109 participants.

Table 3 shows the correlations of age and education with different performance metrics (discussed below). There was a monotonic increase in CRT latencies with age (*r* = 0.46 (range 0.41–0.50), *t*_(1464)_ = 19.84, *p* < 0.0001, slope 2.80 ms/year), and no significant influence of education (*r* = −0.05, NS). Intrasubject CRT variance also increased with age (*r* = 0.17, *t*_(1464)_ = 6.60, *p* < 0.0001). However, because there was a greater age-related increase in CRT latencies than in intrasubject CRT variance, the coefficient of variation (CV, i.e., the Intrasubject CRT variance divided by the participant’s CRT latency) decreased with age (*r* = −0.25 (range −0.20 to 0.30), *F*_(1464)_ = 98.20, *p* < 0.0001).

**Table 3:** **Correlation matrix for Experiment 1**.

	AGE	EDU	RT	RTSD	CV	CPT	AR-CRT	AR-CPT	L-mSOA	Omni
EDU	0.01
RT	0.46	−0.05
RTSD	0.17	−0.02	0.59
CV	−0.25	0.02	−0.09	0.74
CPT	0.42	−0.04	0.93	0.59	−0.03
AR-CRT	0.00	−0.06	0.89	0.57	−0.02	0.83
AR-CPT	0.00	−0.04	0.81	0.57	0.04	0.91	0.92
L-mSOA	−0.02	−0.11	−0.09	−0.10	−0.06	−0.10	−0.10	−0.10
Omni	−0.01	−0.12	0.59	0.36	−0.06	0.54	0.67	0.60	0.67
SRT	0.24	−0.05	0.49	0.18	−0.17	0.13	0.42	0.03	−0.01	0.31

*SRT = simple reaction time (from Woods et al. (2015). See Tables 1, 2 for an explanation of other abbreviations. Given the sample size (*N* = 1466), correlations with |r| > 0.09 are statistically significant following Bonferroni correction (*p* < 0.002)*.

**Figure 3:**
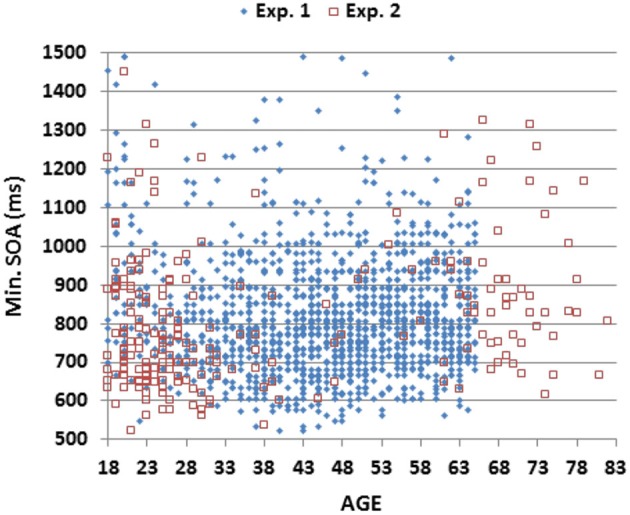
**Minimum stimulus onset asynchronies (mSOAs)**. Shown as a function of age for subjects in Experiments 1 and 2.

As shown in Figure 3; Table 2, mSOAs (mean 825.2 ms) varied non-monotonically with Age-Group (*F*_(6,1462)_ = 11.49, *p* < 0.005, partial *ω*^2^ = 0.04). Further analysis showed that mSOAs were significantly elevated in the youngest and oldest participants, relative to other age groups. mSOA distributions were positively skewed (skew = 2.68) and were therefore log-transformed before further statistical analysis. Log-mSOAs did not correlate significantly with age (*r* = −0.02).

Hit rates averaged 93.5%, with more commission errors (6.5%) than omission errors (2.7%). Hit rate was negatively correlated with mSOA (*r* = −0.55 (range −0.51 to −0.59), *t*_(1464)_ = −25.2, *p* < 0.0001). There was a small but significant Age-Group effect on hit-rate (*F*_(6,1462)_ = 6.57, *p* < 0.0001, partial *ω*^2^ = 0.02), reflecting a lower hit-rate in the youngest age group (91.7%) in comparison to all other age groups (range 93.3%–94.1%), without significant differences between the other age groups. Correlation analysis confirmed the small increase in hit rate with age (*r* = 0.16 (range 0.11–0.21), *F*_(1464)_ = 37.11, *p* < 0.0001). Hit rates correlated positively with CRT latencies (*r* = 0.32 (range 0.27–0.36), *t*_(1464)_ = 12.47, *p* < 0.0001). A multiple regression analysis with Age and Hit-rate as factors showed that CRT latencies were independently influenced by both factors (age, *t*_(1463)_ = 19.02, *p* < 0.0001; hit rate, *t*_(1463)_ = 8.10, *p* < 0.0001).

**Figure 4:**
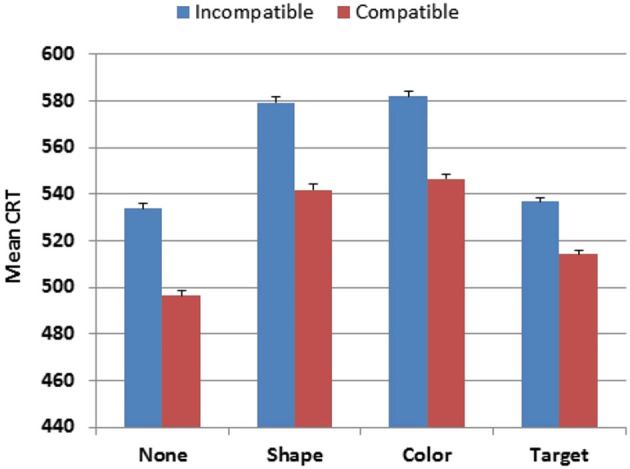
**CRTs to stimuli of different types in Experiment 1**. None = distractor with no target features. Shape = distractor with target shape. Color = distractor with target color. Compatible = stimulus delivered to the visual field ipsilateral to response button. Incompatible = stimulus delivered to the visual field contralateral to the response button. Error bars show 95% confidence intervals.

Stimulus-type also had a strong influence on CRT latencies (*F*_(3,4386)_ = 821.18, *p* < 0.0001, partial *ω*^2^ = 0.36), as shown in Figure 4. Responses were faster to the distractor with no target features (527 ms) and to the target (537 ms) than to distractors with target-shape (573 ms) or distractors with target-color (577 ms). Hit-rate measures showed similar influences of Stimulus-type (*F*_(3,4386)_ = 200.39, *p* < 0.0001, partial *ω*^2^ = 0.12), with more accurate responses to stimuli with no target features (96.3% correct) than to other stimulus types (range 92.5%-92.9%). Power analysis showed a 99% probability of detecting a Stimulus-type effect on hit-rate at the *p* < 0.05 level in 56 participants. The Age-group × Stimulus-type interaction was also significant, but with very small effect sizes for both CRT latencies (*F*_(18,4386)_ = 3.10, *p* < 0.0001, partial *ω*^2^ < 0.01) and hit-rate (*F*_(18,4386)_ = 2.29, *p* < 0.002, partial *ω*^2^ < 0.01), reflecting the slightly larger absolute changes (but similar proportional changes) of CRT latencies and hit-rate in older participants.

Visual field exerted a strong influence on CRT latencies (*F*_(1,1462)_ = 437.80, *p* < 0.0001, partial *ω*^2^ = 0.23): participants were 34 ms faster to respond to stimuli in the visual field ipsilateral to the middle finger (i.e., the right visual field in right-handed participants), presumably reflecting in part the increased probability (60%) of middle-finger responses. There was also a strong Visual-field × Stimulus-type interaction (*F*_(3,4386)_ = 247.54, *p* < 0.0001, partial *ω*^2^ = 0.14) that reflected spatial compatibility effects: responses were faster to stimuli presented in the visual field ipsilateral to the finger used for responding (e.g., the left visual field for targets and the right visual field for distractors in right-handed participants).

In order to examine the effects of spatial compatibility between stimuli and responses, we performed another ANOVA with Visual field spatially compatible or incompatible with the finger used for responding (e.g., the left visual field is spatially compatible with index-finger responses in participants who controlled the mouse with their right hand, and middle-finger responses in participants who controlled the mouse with their left hand). This analysis revealed a large spatial-compatibility effect on CRT latencies (*F*_(1,1442)_ = 1443.34, *p* < 0.0001, partial *ω*^2^ = 0.50), with power analysis showing a 99% probability of detecting a spatial-compatibility effect on CRT latencies at the *p* < 0.05 level in 20 participants. There was a similar spatial-compatibility effect on hit-rate (*F*_(1,1462)_ = 744.54, *p* < 0.0001, partial *ω*^2^ = 0.34), due to higher hit-rates when stimuli were presented in the visual field ipsilateral to the responding finger. Spatial-compatibility effects were slightly larger for distractors than target stimuli for CRT latency (*F*_(3,4386)_ = 16.76, *p* < 0.0001, partial *ω*^2^ = 0.01) and hit-rate (*F*_(3,4386)_ = 15.87, *p* < 0.0001, partial *ω*^2^ = 0.01). Finally, the magnitude of spatial-compatibility effects increased slightly with age on both CRT latencies (*F*_(6,1462)_ = 3.85, *p* < 0.008, partial *ω*^2^ < 0.01) and hit-rate (*F*_(6,1462)_ = 2.28, *p* < 0.05, partial *ω*^2^ < 0.01), but with small effect sizes.

There were several negative results of note. Sex differences in CRT latencies failed to reach significance (*F*_(1,1467)_ = 0.33, NS), nor were there significant sex differences in CRT SDs (*F*_(1,1467)_ = 1.06, NS). However, mSOAs were slightly shorter in female participants (819 vs. 841 ms, *F*_(6,1462)_ = 4.7, *p* < 0.05, partial *ω*^2^ < 0.01). Education had no significant influence on CRT latency (*r* = −0.05), hit rates (*r* = 0.05), standard deviations (*r* = −0.02), or CV (*r* = 0.02). However, increased education was associated with a small reduction in mSOA (*r* = −0.10, *F*_(1464)_ = 13.59, *p* < 0.0001).

**Figure 5:**
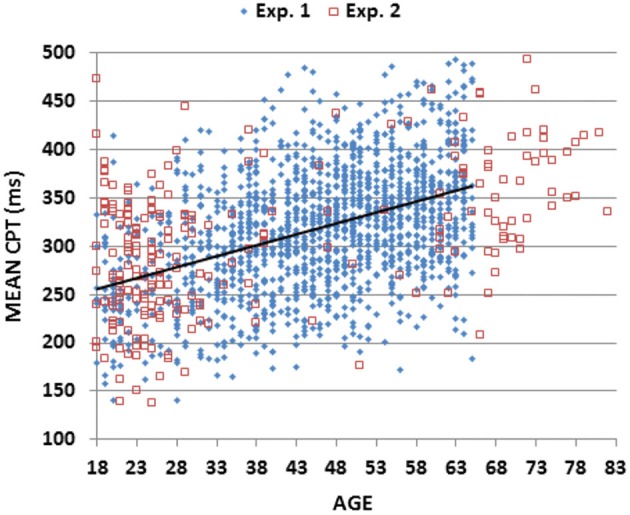
**Mean central processing time (CPT)**. CPTs were derived by subtracting RT latencies in a simple reaction time (SRT) task from CRT latencies averaged over different stimuli for subjects of different ages in Experiments 1 and 2. Linear fit is shown for Experiment 1 data.

As shown in Table 3, simple reaction times (SRTs) measured in a companion study (Woods et al., 2015) were strongly correlated with CRT latencies in the current experiment (*r* = 0.49 (range 0.44–0.53), *t*_(1464)_ = 21.52, *p* < 0.0001). We obtained a measure of Central Processing Time (CPT) by subtracting SRTs from CRT latencies. As shown in Figure 5 (blue diamonds), CPTs (mean 319.1 ms) showed a monotonic increase with age (slope 2.26 ms/year, *r* = 0.42 (range 0.37–0.47), *t*_(1464)_ = 17.71, *p* < 0.0001), and varied significantly across Age-Groups (*F*_(6,1462)_ = 50.05, *p* < 0.0001, partial *ω*^2^ = 0.17) without differences between the sexes (*F*_(1,1467)_ = 0.00, NS).

A comparison of the age slopes for CPTs and CRT latencies suggests that 82% of overall age-related CRT slowing reflected age-related slowing in the CPT. We used additional subtraction procedures to analyze age-related changes in the time required for different processing stages. CRT latencies in the current experiment reflected the time needed to (1) detect the stimulus; (2) identify the stimulus and select an appropriate response; and (3) depress the button. Previous SRT and finger-tapping studies of the same participants in the same test session had provided estimates of stage 1, stimulus-detection time (SDT; Woods et al., 2015), and stage 3, movement initiation time (MIT; Hubel et al., 2013a).

The time required for stage 2 was reflected in the CPT: i.e., the difference between CRT and SRT latencies. The CPT was further conceptually subdivided into a minimal CPT (MCPT) stage and continued feature processing time (CFPT). The duration of the MCPT was quantified as the difference in CRT latencies to distractors with no target features and SRTs in each participant. The MCPT stage would therefore include the time needed to discriminate the distractor with no target features from the target (relative to the time needed to merely detect the occurrence of a stimulus in the SRT task), as well as the time needed to select the appropriate response. The duration of the CFPT was estimated from the additional time (mean 47.4 ms) required to identify distractors with target color or shape relative to distractors with no target features.

**Figure 6:**
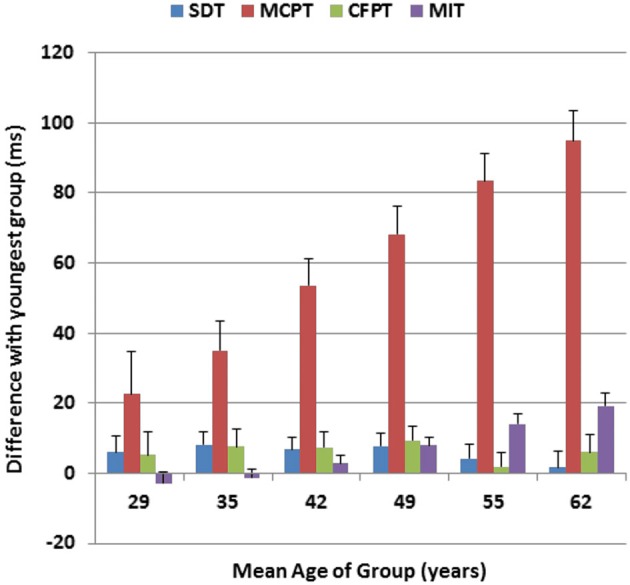
**Age-related changes in different processing stages for Experiment 1**. Changes in ms are shown relative to the duration of each processing stage in the youngest subjects (18–24 years). Stimulus detection time (SDT) and movement-initiation time (MIT) had been measured in previous tests performed on the same day. MCPT = Minimal CPT, the difference between CRTs to distractors with no target features and simple reaction times. CFPT = continued feature processing time, the difference between CRTs to distractors with target color or shape and distractors with no target features. Error bars show 95% confidence intervals.

Figure 6 shows the age-related changes in the duration of each of the four processing stages, relative to their durations in the youngest participant group. As previously described (Woods et al., 2015), SDT was not significantly affected by age, while MIT showed a linear 20% increase with age (Hubel et al., 2013a). The MCPT stage was strongly correlated with age (*r* = 0.40, *t*_(1464)_ = 16.70, *p* < 0.0001), and showed a gradual and monotonic increase to the point that the duration of this stage was lengthened by 40% in the oldest participant group. In contrast, the duration of the CFPT stage was not significantly correlated with age (*r* = 0.01). Of the total age-related CRT slowing (120.6 ms), roughly 80% was due to delays in the MCPT, 16% was due to slowed MITs, and less than 4% of the slowing was due to delays in stimulus processing in the SDT or CFPT stages.

Given the large influence of age on CRT latencies, age-regressed CRT norms (AR-CRTs) were calculated using age-predicted CRT latencies based on the regression equation: CRT = 422 + (Age)*2.80. AR-CRTs had a residual standard deviation of 64.4 ms. Age-regressed CPTs (AR-CPTs) were similarly calculated using the regression equation: CPT = 216 + Age*2.26, resulting in a residual standard deviation of 57.9 ms. Finally, to quantify overall performance on the visual feature conjunction task for comparison with other populations, we created an Omnibus *z*-score by combining AR-CRT *z*-scores and log-transformed mSOA *z*-scores from each participant.

## Discussion: Experiment 1

The increase in CRT latencies with age in the current study (2.8 ms/year) was similar to increases previously reported in the studies of age-related changes in CRT latencies included in Table 1. Highly significant effects of aging were found: mean CRT latencies in 59–65 year old participants were increased by more than 120 ms (1.8 standard deviations), with respect to the youngest participant group.

Greater age-related slowing was found when participants performed CRT tasks compared to SRT tasks: the correlation between age and CRT latencies was significantly stronger than the correlation between age and SRTs (*r* = 0.46 vs. *r* = 0.24, *z* = 6.55, *p* < 0.0001) that was measured in the same participants in a previous study (Woods et al., 2015). Age-related CRT slowing was also proportionally larger than SRT slowing: CRT latencies increased by 25.1% in the oldest participant group, whereas SRT latencies increased by only 9.6%. This is in agreement with many studies that find larger absolute and relative age-related increases in CRT latencies than SRTs (Yordanova et al., 2004; Anstey et al., 2005; Deary and Der, 2005; Deary et al., 2010; Era et al., 2011). Finally, The magnitude of CRT slowing was similar to the age-related increases in search asymptotes observed in both feature search and conjunction search conditions (Plude and Doussard-Roosevelt, 1989; Hommel et al., 2004; Müller-Oehring et al., 2013).

In accord with previous studies, we found that aging increased both inter-subject standard deviations (Bugg et al., 2006; Vincent et al., 2012) and intrasubject (trial-to-trial) standard deviations (Deary and Der, 2005; Reimers and Maylor, 2006). However, in contrast to previous studies (Dykiert et al., 2012a), older participants in the current study showed reduced CVs. Among the many methodological differences between previous large-scale studies and the current experiment, we gathered data from more trials and provided more training, suggesting that older participants may require a longer training period to achieve stable performance. We found no sex differences in CRT latencies, nor did we find significant sex differences in intrasubject reaction time variance or hit-rate. These results also agree with some previous studies (Deary and Der, 2005), but conflict with others (Houx and Jolles, 1993; Dykiert et al., 2012b).

Subtraction analysis showed that 80% of the age-related slowing in the current experiment was due to slowed MCPTs, the difference between CRT latencies to distractors with no target features and SRT latencies. The remaining age-related delay was due largely to slowed motor responses. Plude and Doussard-Roosevelt (1989) found that older participants required 49.6 ms/item in feature-conjunction conditions, and younger participants required 25.5 ms/item. Thus, their reported age-related increase in the time needed to integrate visual features (e.g., 24.1 ms/item) would account for about 25% of the age-related increases (120.6 ms) in MCPT. Although participants of all ages took longer to discriminate target-like distractors (i.e., with target color or shape) than distractors with no target features, CFPT (the difference in CRT latencies of these stimuli and distractors with no target features) increased by only 4 ms in older participants. This suggests that any increase in feature-integration time in older participants was largely independent of the features that were combined, and of the resemblance of the distractor to the target.

Unlike SRTs, which differ by less than 4 ms for stimuli presented in the two visual fields (Woods et al., 2015), CRT latencies showed large visual-field effects both for reaction time and hit-rate that largely reflected the spatial compatibility of the stimuli and responses (Klein and Ivanoff, 2011). As in previous studies (van der Lubbe and Verleger, 2002; Linnet and Roser, 2012), we found that spatial compatibility effects increased with age. Age-related increases in compatibility effects are consistent with the suggestion that the response-selection process is affected by aging. This hypothesis is also supported by ERP studies, which find significant age-related delays in the time between the onset of motor cortex activation and the onset of EMG activity in the muscles executing the response (Yordanova et al., 2004; van de Laar et al., 2012).

## Experiment 2: A Large-Scale Replication

In addition to examining aging effects, an ancillary goal of Experiment 1 was to develop a reliable CRT test for clinical use. CRT tests have been incorporated into a number of computerized cognitive test batteries (Lee et al., 2005; Lapshin et al., 2013), including the Automated Neuropsychological Assessment Metrics (ANAM; Kane et al., 2007), Cambridge Neuropsychological Test Automated Battery (CANTAB; Robbins et al., 1994), the Immediate Post-Concussion Assessment and Cognitive Testing battery (ImPact) (Iverson et al., 2005; Maerlender et al., 2010; Allen and Gfeller, 2011), and the CNS Vital Signs Computerized Neurocognitive Test (CNS) (Gualtieri and Johnson, 2008). These tests are commonly used to detect age-related cognitive impairments (Rabbitt et al., 2007; Luciano et al., 2009; Ballesteros et al., 2013), but lack normative data obtained from multiple, large-scale data sets.

Previous large-scale studies of age-related changes in CRT latencies have reported widely varying results at different sites. For example, studies using a reaction time box in which participants press one of four buttons in response to the digits 1–4 (see Table 1) have produced CRT latencies ranging from 518 ms (Dykiert et al., 2012b) to 728 ms (Deary et al., 2001) in participants of similar age ranges, with age slopes ranging from 2.2 to 3.4 ms/year. In Experiment 2, we investigated how accurately the age-regression functions from participants tested in Experiment 1 in New Zealand would fit the results of 178 control participants with different demographic backgrounds tested in Northern California.

## Methods: Experiment 2

The methods were identical to those in Experiment 1.

### Participants

We recruited 178 participants from the San Francisco Bay Area through internet advertisements and from existing control participant populations. Their demographic characteristics are summarized in Table 2. All participants signed written consent forms approved by the institutional review boards (IRB) at the Veterans Affairs Northern California Health Care System (VANCHCS) and were paid for their participation. Participants were required to meet the following inclusion criteria: (a) fluency in the English language; (b) no current or prior history of bipolar disorder, mania, or schizophrenia; (c) no current substance abuse; (d) no concurrent history of neurologic disease known to affect cognitive functioning; (e) on a stable dosage of any required medication; (f) auditory functioning sufficient to understand normal conversational speech and visual acuity normal or corrected to 20/40 or better. Participant ethnicities were 61% Caucasian, 11% African American, 12% Asian, 10% Hispanic/Latino, 2% Hawaiian/Pacific Islander, 2% American Indian/Alaskan Native, and 3% “other.” Participants underwent CRT testing midway through the two hour California Cognitive Assessment Battery (CCAB), a set of computerized neuropsychological tests and questionnaires.^1^

Unlike the participants in Experiment 1, who had been recruited as a community sample with balanced age distributions, the age distribution of Experiment 2 was largely bimodal: 90 participants were below the age of 30 years, 30 participants were between the ages of 30 and 59 years, and 58 participants were between the ages of 60 and 82 years. As a result, the mean age of participants in Experiment 2 was slightly lower than that of participants in Experiment 1 (41.2 vs. 45.8 years, *t*_(1656)_ = 4.70, *p* < 0.0001). The participants were predominantly male (58%) and better educated than the participants in Experiment 1 (14.7 vs. 12.5 years of education, *t*_(1656)_ = 8.64, *p* < 0.0001). The 45 participants over the age of 65 years were particularly well-educated (mean 15.1 years of education).

### Timing Calibration

Identical computer hardware and software was used in the two testing laboratories so that device-specific hardware delays were identical to those in Experiment 1. The median timing uncertainty of 60,166 events was 0.1 ms (range 0.1–41.5 ms), with 99.9% of events showing timing uncertainties less than 0.8 ms.

### Data and Statistical Analysis

Procedures were identical to those used in the previous experiment. Age-regressed *z*-scores were calculated using the values obtained in Experiment 1.

## Results: Experiment 2

**Figure 7:**
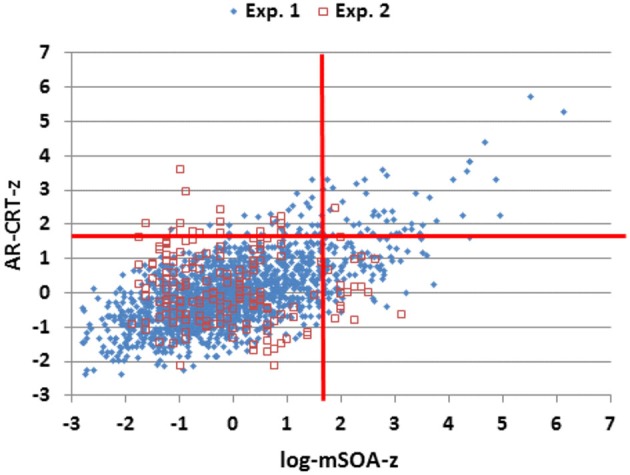
***Z*-scores of age-regressed (AR) CRTs and log-mSOAs**. *Z*-scores were calculated based on means and age-regression slopes from Experiment 1 data for individual subjects in Experiments 1 and 2. The abnormal performance thresholds (red lines, *p* < 0.05) were derived from Experiment 1 data.

The results of Experiment 2 are summarized in the rightmost column of Table 2. Mean CRT latencies differed by 4 ms from those obtained in Experiment 1, and overlapped the range of values seen in Experiment 1, as shown in Figures 2, 5 (open red squares). The performance of participants in Experiment 2 was well-fit by the regression functions from Experiment 1, as shown in the scatter plot of age-regressed (AR-) CRT latencies and log-mSOA *z*-scores in Figure 7. CRT latencies were 9.5 ms above age-predicted values, resulting in an insignificant difference in *z*-scores between the two experiments (mean AR-CRT *z*-score = 0.15, *F*_(1,1642)_ = 3.38, *p* < 0.07). Mean mSOAs were 8 ms below those in Experiment 1, and log-mSOA *z*-scores did not differ significantly between the two experiments (*z*-score = −0.08, *F*_(1,1642)_ = 0.77, NS). Finally, omnibus *z*-scores (0.08) for combined CRT latencies and log-mSOAs did not differ significantly between the two experiments (*F*_(1,1642)_ = 0.93, NS).

Stimulus-type and stimulus-response compatibility effects replicated those seen in Experiment 1. CRT latencies were faster to stimuli with no target features (529 ms) and targets (527 ms) than to distractors with target-shape (571 ms) or color (576 ms) (*F*_(3,531)_ = 101.42, *p* < 0.0001, partial *ω*^2^ = 0.36). As in Experiment 1, responses were also more accurate for stimuli with no target features (95.8% ms) than to other stimulus types (range 92.2% to 92.6%, *F*_(3,531)_ = 23.10, *p* < 0.0001, partial *ω*^2^ = 0.11). CRT latencies were shorter to spatially compatible stimuli (by 33 ms, *F*_(1,177)_ = 167.12, *p* < 0.0001, partial *ω*^2^ = 0.48), and spatial compatibility also exerted significant effects on hit-rate (95.5% vs. 91%, *F*_(1,177)_ = 128.26, *p* < 0.0001, partial *ω*^2^ = 0.42).

Table 4 shows the correlation matrix for Experiment 2. There was a strong correlation between age and CRT latencies (*r* = 0.52 (range 0.39–0.65), *t*_(176)_ = 8.08, *p* < 0.0001) and age and CPTs (*r* = 0.48 (range 0.34–0.61), *t*_(176)_ = 7.06, *p* < 0.0001). Age-related increases in the CPT (1.57 ms/year) accounted for more than 80% of the age-related slowing in CRT latencies (1.91 ms/year). Most of the remaining age-related slowing reflected increases in MIT (*r* = 0.42 (range 0.28–0.55), *t*_(176)_ = 6.14, *p* < 0.0001). As in Experiment 1, the MCPT was strongly correlated with age (*r* = 0.41 (range 0.28–0.55), *t*_(176)_ = 5.94, *p* < 0.0001), while CFPT did not show a significant age correlation (*r* = 0.12, *t*_(176)_ = 1.60, *p* < 0.12).

**Table 4:** **Correlation matrix for Experiment 2**.

	AGE	EDU	RT	RTSD	CV	CPT	AR-CRT	AR-CPT	L-mSOA	Omni-Z
EDU	0.17
RT	0.52	−0.01
RTSD	0.12	−0.07	0.58
CV	−0.21	−0.07	0.00	0.81
CPT	0.48	−0.01	0.96	0.60	0.05
AR-CRT	−0.27	−0.15	0.68	0.55	0.19	0.67
AR-CPT	−0.23	−0.15	0.67	0.57	0.22	0.75	0.95
L-mSOA	0.26	−0.04	0.09	−0.03	−0.11	0.11	−0.11	−0.07
Omni-Z	−0.03	−0.16	0.59	0.40	0.06	0.59	0.69	0.68	0.63
SRT	0.31	0.02	0.48	0.16	−0.15	0.20	0.27	−0.01	−0.01	0.21

*See previous tables for an explanation of abbreviations. Given the sample size (N = 178), correlations with |r| > 0.21 are statistically significant following Bonferroni correction (p < 0.002)*.

However, there were several significant differences between the Experiments, as seen in the comparison of the correlation matrices in Tables 3, 4. Unlike Experiment 1, log-mSOAs in Experiment 2 increased with age (*r* = 0.26 (range 0.11–0.40), *t*_(176)_ = 3.57, *p* < 0.0005). This reflected the inclusion of participants over 65 years of age. When these participants were excluded, the Age vs. log-mSOA correlation fell to insignificance (*r* = 0.11, *p* < 0.11). As in Experiment 1, there were no significant sex differences in CRT, intraparticipant CRT variance, or CV, and female participants had slightly shorter mSOAs (by 59 ms, *t*_(176)_ = 2.20, *p* < 0.03).

In addition, the negative correlation between age and AR-CRTs in Experiment 2 (*r* = −0.27) was significantly different from the null correlation in Experiment 1 (*z* = 3.46, *p* < 0.0005). This indicates that the older participants in Experiment 2 had relatively faster CRT latencies than predicted from Experiment 1 data. In contrast, log-mSOAs showed an insignificant correlation with age in Experiment 1 (*r* = −0.03), but a significant positive correlation in Experiment 2 (*r* = 0.26), resulting in a significant difference (*z* = −3.58, *p* < 0.0005). These results suggest that the younger participants in Experiment 2 placed a greater emphasis on accuracy than the younger participants of Experiment 1, whereas the older participants in Experiment 2 placed a greater emphasis on speed than did their counterparts in Experiment 1.

## Discussion

Large scale replications of CRT tasks are relatively rare. While commercial CRT tests (e.g., Cantab) have not reported the results of multiple large scale normative studies, large-scale non-commercial CRT paradigms have obtained widely different mean CRT latencies using apparently identical test procedures (Table 1). For example, using a similar four-choice CRT paradigm, Deary et al. (2001) found mean CRT latencies of 728 ms in a large group of 56 year old participants, while Deary et al. (2011) found mean CRT latencies of 556 ms in participants with a mean age of 48 years. These differences were substantial: the mean CRT latencies reported in Deary et al. (2001) were increased by 172 ms, nearly 2.0 standard deviations, in comparison with the results of Deary et al. (2011). Moreover, Dykiert et al. (2012b) used an identical paradigm in a population with a mean age of 40 years, and obtained mean CRT latencies that were 210 ms shorter than those obtained by Deary et al. (2001); i.e., a difference much greater than the 32–48 ms that would be predicted based on the different mean ages of the participant populations.

The sources of imprecision in previous large-scale CRT studies remain obscure. Differences in computer hardware and software can alter measured CRT latencies by more than 100 ms (Neath et al., 2011), even with modern computer systems. This suggests that some of the variation in CRT latencies may reflect differences in the timing precision of the digital systems used for CRT measurement (Plant and Quinlan, 2013). In addition, Dordonova and Dordonov (2013) argued that temperature sensitivity may have altered the display delays in the response box systems used to measure CRT latencies by Deary and colleagues.

We used carefully calibrated computer hardware and software that introduced minimal timing delays and found a difference of only 4 ms in mean CRT latencies in large subject populations that were tested independently in New Zealand and California. Participants at both sites produced similar AR-CRT *z*-scores, log-mSOA *z*-scores, and Omnibus *z*-scores. This suggests that, given precise computer software and appropriate hardware timing calibration, the normative data and age-regression functions obtained in one laboratory can be used to accurately evaluate the performance of participants tested at other sites.

The principle findings of Experiment 2 replicated those of Experiment 1. The majority of the age-related increases in CRT latency reflected age-related increases in the CPT, due primarily to increases in the MCPT. As in Experiment 1, no significant age-related increases were found in CFPT (i.e., the CRT difference between target-like distractors and distractors with no target features). As the MCPT showed strong stimulus-response spatial-compatibility effects, the results suggest that the majority of age-related slowing seen in CRT tasks is due to slowed feature integration and response selection.

The differences in the results of the two experiments were relatively minor. The small cohort of older (>65 years), well-educated participants in Experiment 2 apparently emphasized speed over accuracy, producing shorter CRT latencies (and hence, CPTs) than predicted by the age-regression functions of Experiment 1. They also made more errors, as reflected in increased log-mSOAs. This speed/accuracy tradeoff among the older participant cohort is also consistent with the shallower age-slope of CRT latencies in Experiment 2 (1.91 ms/year) than that of Experiment 1 (2.80 ms/year).

In contrast to previous studies (Dykiert et al., 2012a), we did not observe an increase in the CV of trial-to-trial RT variance with age. Indeed, as in Experiment 1, we found that the intrasubject CV actually decreased in older participants in Experiment 2. Similarly, in contrast to some previous studies (Dykiert et al., 2012b), we failed to find significant sex differences in CRT latencies, CRT trial-to-trial variance, or CRT CVs, again replicating the results of Experiment 1.

## Conclusions

Age-related changes in visual CRT latencies were examined in a rapid serial visual feature-conjunction task in two large-scale experiments. Mean CRT latencies in the two experiments differed by only 4 ms, and, in both experiments, CRT latencies increased markedly with age. Most of the age-related increases in CRT latencies reflected delays in CPT, isolated by subtracting SRTs from CRT latencies. Participants were faster when the stimulus location and mouse button were spatially compatible, and the spatial compatibility effect also increased with age. The results suggest that age-related slowing in visual CRT latencies largely reflects delays in response-selection and motor execution.

## Conflict of Interest Statement

DLW is affiliated with NeuroBehavioral Systems, Inc., the developers of Presentation software used to create these experiments.

## Footnotes

^1^The CCAB included the following computerized tests and questionnaires: finger tapping, simple reaction time, Stroop, digit span forward and backward, verbal fluency, verbal list learning, spatial span, trail making, vocabulary, design fluency, the Wechsler Test of Adult Reading (WTAR), visual feature conjunction, risk and loss avoidance, delay discounting, the Paced Auditory Serial Addition Task (PASAT), the Cognitive Failures Questionnaire (CFQ), the Posttraumatic Stress Disorder Checklist (PCL), and a traumatic brain injury (TBI) questionnaire.
