# Corrigendum: Age-related slowing of response selection and production in a visual choice reaction time task

**DOI:** 10.3389/fnhum.2015.00350

**Published:** 2015-06-17

**Authors:** David L. Woods, John M. Wyma, E. William Yund, Timothy J. Herron, Bruce Reed

**Affiliations:** ^1^Human Cognitive Neurophysiology Laboratory, Veterans Affairs Northern California Health Care SystemMartinez, CA, USA; ^2^Department of Neurology, UC DavisSacramento, CA, USA; ^3^Center for Neurosciences, UC DavisDavis, CA, USA; ^4^Center for Mind and Brain, UC DavisDavis, CA, USA; ^5^Alzheimer's Disease CenterDavis, CA, USA

**Keywords:** gender, timing, processing speed, motor, handedness, hemisphere, replication, executive function

The x axis in Figure 7 was mislabeled as log-mSOA-z instead of Omnibus z.

**Figure 7:**
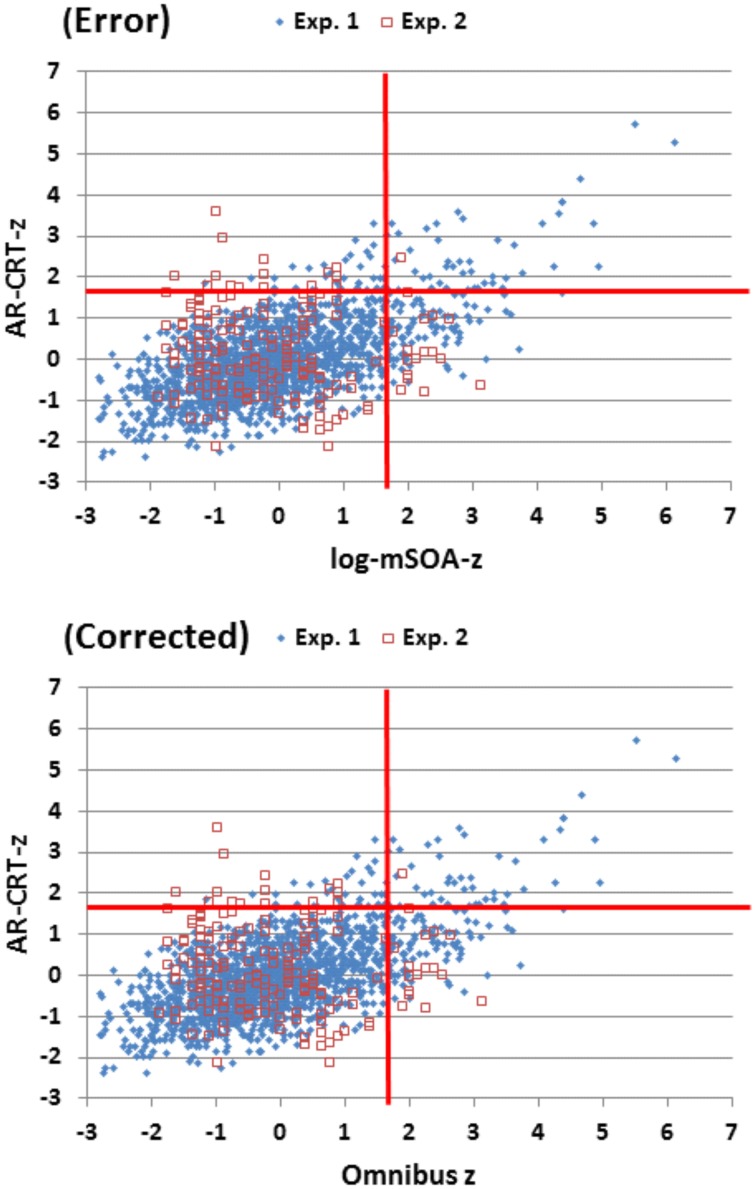
**Z-scores of age-regressed (AR) CRTs and omnibus scores**. Z-scores were calculated based on means and age-regression slopes from Experiment 1 data for individual subjects in Experiments 1 and 2. The abnormal performance thresholds (red lines, *p* < 0.05) were derived from Experiment 1 data.

## Conflict of interest statement

DW is affiliated with NeuroBehavioral Systems, Inc., the developers of Presentation software used to create these experiments. The authors declare that the research was conducted in the absence of any commercial or financial relationships that could be construed as a potential conflict of interest.

